# Reflex Epilepsy1Read before the Section on Medicine of the Buffalo Academy of Medicine.

**Published:** 1895-11

**Authors:** William C. Krauss

**Affiliations:** Buffalo, N. Y., Professor of nervous diseases, Medical Department Niagara University; neurologist to the Erie County Hospital.


### REFLEX EPILEPSY.[1]

By WILLIAM C. KRAUSS, M. D., Buffalo, N. Y.,
Professor of nervous diseases, Medical Department Niagara University; neurologist to the Erie County Hospital.

IN A paper read before the eighty-seventh annual meeting of the Medical Society of the State of New York, on Reflex disturbances in the causation of epilepsy, I entered into the mechanism of reflex epilepsy at some length and am still satisfied, in my own mind, that the etiology there given is as nearly correct as we are at present able to pronounce. I shall quote frequently from that paper this evening, amending and modifying only where my views have undergone a change.

Reflex epilepsy is certainly the most amenable to treatment of any of the various kinds of epilepsy, and hence it deserves careful consideration to be able to distinguish it. Before discussing the etiology of reflex epilepsy, it might be well to know what we mean by epilepsy. I would describe epilepsy as a complex symptom arising from a multiplicity of causes, having for its background a decided neurotic disposition, either inherited or acquired. Reflex epilepsy is that form where the direct causative agent may be traced to some peripheral nerve irritation. The mechanism of such irritation acting upon the brain, causing epileptic convulsions, may be described thus:

We learn by studying the fundamental laws of nature that all matter is in a constant state of motion, and anything that hastens or retards this vibration produces an equivalent amount of heat or heat values. Moreover, that force cannot be annihilated. If destroyed in one form it reappears in another, without suffering any loss. Apply these laws to the human system and we can account for reflex epilepsy perhaps as follows: a perfectly normal nervous system holds sway over a healthy, vigorous organism; the functions of secretion, excretion and assimilation follow closely the physiological laws governing them. The result of these metabolic forces means work, either mental or physical, or both, along some clearly defined channel. The whole system, then, is in a normal state of motion, each fiber and gland working faithfully and assiduously to keep the structure in a state of perfect equilibrium. Perchance a nerve trunk or nerve terminal is subjected to some insult causing obstruction, retardation or interference of its molecular vibration; accordingly, heat units or heat

1. Read before the Section on Medicine of the Buffalo Academy of Medicine.

values are generated because of this irritation, reflected to the great heat center, the brain, and these converted into heat equivalents such as pain, tremor, spasm, and the like, referable to the seat of the initial lesion. This process continuing indefinitely without relaxation, accumulation of heat values ensue and an explosion of nerve force is the result. Reflex epilepsy means, therefore, a state of irritation of the cerebral centers, produced not by a central but by a peripheral lesion.

The line of demarkation between reflex and traumatic epilepsy should be sharply drawn. In the latter the irritative lesion is direct and local, the result of a trauma to the cranium, and the consequent convulsions are in a degree amenable to the surgeon's art, yet not to such an extent as was claimed by observers a few years ago.

It is true that not every scar, ingrown toe-nail, phimosis, pin-worm, or what not, begets reflex cerebral disturbance, or else this form of disease would be as wide-spread and prevalent as was the itch during the middle ages. Therefore some other property or idiosyncrasy must be present in order that the conditions be fulfilled. A neuropathic disposition, either inherited or acquired, is the *sine qua non* of this symptom-complex. This need not necessarily be present at the time the irritation commences, but may be saddled upon the system, the result of the tireless, unceasing effort of the brain to counteract the nerve strain. The patient in a short time becomes peevish, excitable, anemic, and the parents will state that the child has of late "become nervous." In those cases of an inherited neurotic disposition the convulsive attacks appear much sooner after the advent of the irritative lesion and, as a rule, the epileptic habit is early and fast ingrafted upon the constitution. This irritation once relieved or remedied, the habit still persists in these latter cases, and to all intents and purposes the patient has the appearance of suffering with idiopathic epilepsy. When such is the case, the nature and origin of the disease is almost impossible to ascertain and the epilepsy is erroneously classified as spontaneous or inherited.

The question may now be asked, What shall we look for in deciding the immediate causation of reflex epilepsy? As a rule, whenever practicable, I have the patient remove all his clothing and examine him thoroughly, beginning with the feet.

Ingrown toe-nails, corns and callouses are not infrequently the cause of epilepsy. Scars about the limbs, disorders of the genitalia, incomplete descent of one or both testes, are some of the causes in youth. In girls the condition of the clitoris and mouth of the vagina should be carefully ascertained ; also whether there is incontinence of urine or the presence of oxyurides in the vagina or rectum. Hare, in his monograph on epilepsy, says "that it may be laid down as a fact that in all cases in girls, in which epilepsy of unknown cause develops, the vagina should be examined for the presence of any pinworms which may have emigrated from the rectum." The condition of the rectum should not be overlooked, but carefully explored for ulcers and irritating hemorrhoids. Coming to the head we have an extensive field for examination. The mouth, nose, ears, eyes and scalp may all harbor seemingly trivial disturbances, which under other circumstances would pass unheeded and unnoticed. Disorders of dentition, such as faulty direction in the growth of the teeth, hidden fangs, caries, neoplasms in and about the buccal cavity, retained foreign bodies in the pharynx, scars, the result of ulcerative or specific processes about the tonsils, tongue and larynx, polypi and foreign bodies in the nose and ears, disorders of refraction ; in short, whatever can impinge, either directly or indirectly, upon nerve trunks or nerve filaments, other conditions being equal, may produce reflex symptomatic epilepsy. These, then, are some of the external causes, and to discover them is easy, very easy, as compared with the probable internal causes. No doubt you are all acquainted with the stomach as a very frequent contributor to this subject. Those of us who pay special attention to nerve diseases receive many cases labeled epilepsy due to stomach trouble. The round worms of the intestines are a common cause and have been recognised as one of the most prolific agents in the etiology of this disease. Disturbances of nearly every internal organ have, according to various writers on this subject, at one time or another produced epilepsy.

There are three organs which observers declare are most frequently associated with this form of epilepsy—the stomach, eyes and penis. The importance of *the stomach* as a primary etiological factor in the causation of reflex epilepsy is, in my opinion, overestimated. I will not deny that the ingestion of indigestible matter invites convulsions, and that after a brisk purge or emesis they disappear. Many patients tell their physician that such was the cause, the starting point of the first attack, and the physician confidingly tells the specialist the same story. They will further testify that they can foretell the approach of an attack by the voracious appetite, peculiar sensation at the pit of the stomach, vomiting of watery fluid and the patient's desire to eat *anything* and *everything* that comes within his reach. Put this patient on a bland or exclusive milk diet and keep him on it for months, give him pepsin, trypsin, papoid and the like, and if your experience is similar to mine, he will continue to convulse and froth at the mouth, and you are fortunate, indeed, if his parents do not become similarly affected. I believe that in many of these cases we are on the wrong trail and must seek the cause elsewhere. The symptoms denoting gastric affinity are not so much epileptogenic as epileptopathic. These disturbances, as the patient and parents declare, hold a close relation to the paroxysms, and I believe they are only localised epileptic attacks or epileptic equivalents. The increased tension in which the nervous system is held just prior to an attack, stimulates the gastric glandular system, through the sympathetic and pneumogastric nerves calling forth an abnormal secretion of gastric fluids, and the flow of gastric juice means an appetite. The stomach naturally becomes tender and hyperesthetic under such rule long continued, and anything that irritates or demands undue attention is rewarded with stern rebuke. I have seen the stomach perform similar antics in hysteria and general paresis, and yet neither you nor I would affirm that the gastric trouble was the cause of these ailments.

The refractive anomalies and muscular insufficiencies of the eye have come in for a good share of the reflex patronage. A few years ago, the startling announcement was made by a New York physician that chorea, epilepsy and insanity are functional nervous diseases caused by refractive errors, the relief of which, by glasses, would cure these disorders. A little later, this versatile physician shifted his position, or sufficiently stretched the margins, to include muscular insufficiencies as further causes of these affections—hence, tenotomies and prisms would cure what cylinders and spheres had failed to do. The New York Neurological Society, after a thorough investigation of the above claims, failed to substantiate them ; and D. B. St. John Roosa, in a valuable paper entitled The relation of errors of refraction and insufficiencies of the ocular muscles to functional diseases of the nervous system, completely exploded this theory. He states :

From an examination of 6,455 eye cases, defective muscular or refractive states do not necessarily produce even *local* disturbances, such as are comprehended under the term asthenopia, inflammation of the edges of the lids, and the like, although high degrees of hyperme-tropia, and moderate degrees of astigmatism, and all cases of mixed astigmatism *are apt to do so*, sooner or later.

He further remarks that asthenopia depends chiefly upon two sets of causes, nervous exhaustion and uncorrected errors of refraction. You observe he places a constitutional or general cause first.

In the *Lancet* of October 28, 1893, H. W. Dodd reports 100 consecutive cases of epilepsy, their refraction and their treatment by glasses, in which he states that of the 100 cases 25 did not require glasses, so they were not prescribed. Of the remaining 75 cases, only 52 secured and wore glasses as ordered. Of these 52, he says, 13 have had no fits since wearing the glasses during periods varying from one year to four months. Three cases remained in *statu quo* and 36 improved in a marked degree. Dodd summarises his views as follows: "Given a certain condition of unstability of the nervous system, (*a*) errors of refraction may excite epilepsy ; (*b*) the correction of the errors of refraction will, in combination with other treatment, in many cases, cure or relieve the epileptic condition."

The reprehensible practice of saddling a pair of glasses on every patient who comes into the consultation-room, or of doing a tenotomy for every known ache, pain or grunt, has caused physicians to stop and ask whether the *specialty* business, as practised by some, has not become one of fake, fuss and fiction. The substratum upon which all these cases rest, individual susceptibility, or an acquired or inherited nervous predisposition, is entirely overlooked, and a routine treatment is adopted for the mass of patients, instead of an individualised treatment.

The penis as an important provocative agent has long been recognised, but under the same conditions which underlie the reflex disturbances of the other organs. For instance, the prepuce may be too long or too short, adherent or constricted ; the frenulum too short, producing a mild form of hypospadias ; or the prepuce has never been everted, and large accumulations of smegma are present. Supposing that none of these conditions are found, you have still the most important examination to make—the exploration of the urethra—and how often is it undertaken in these cases ? The boy has never had connection, and, hence, you infer no gonorrhea with its attending inflammation and strictures. But try to pass a No. 12 or 15 Benas catheter ; and, although they glide along so nicely at other times, you are beset with obstacles in passing through constrictions, evading the boy's hands, and in convincing him that his urethra is anesthetic and he cannot have any pain. I am thoroughly satisfied that there are many disturbances present in a good boy's urethra which prompt him to vicious habits; and they, in turn, if his constitution is impressionable, lead to serious nervous disorders, such as chorea, epilepsy, neurasthenia, and the like. Vegetations, polypi, cysts or other neoplasms in the walls of the urethra, producing constrictions, hyperesthetic areas, slow forms of inflammation are translated by such symptoms as priapism, spasm of the urethral muscles, frequent micturition, spermatorrhea, masturbation and sexual excesses. The influence of these morbific agencies upon the nervous system is too well known to be here reviewed. In a short period of time, I have treated six boys with reflex neuroses, five of whom had reflex epilepsy, due to disturbances along the urethral canal. In some of these cases I have found the meatus urinarius surrounded by a red border or ring, portraying, perhaps, the congestive state of the urethral lining. An urethroscopic examination should never be omitted, for by its revelation the exact nature of the lesion can be determined.

To sum up the subject of reflex irritation, I will quote the conclusions of Ayres, in a recent number of the *Pittsburgh Medical Review:*

With reference to reflex irritation, there are three classes of patients:

1. Those persons of normally robust organisations, on whom ordinary irritations play in vain; their equilibrium cannot be disturbed by anything short of a decided cause.

2. Those persons of unstable nervous systems, who are susceptible to reflex disturbances, but from careful avoidance, and other fortunate circumstances, largely escape their effect.

3. Those neuropathic, sensitive constitutions, so delicately balanced that a single depressing or irritating agent will throw them into an abnormal or pathological state.

Gray's conclusions in regard to diseases of the male and female genital organs to mental and nervous diseases are worthy of careful consideration. He says:

1. There is no proof that genital irritation, in the male or female, can cause nervous or mental disease, except in a predisposed individual.

2. That the proof is not yet absolute that genital irritation can produce nervous or mental disease even in a predisposed individual.

3. That there is undoubted proof that the relief of the genital disease, in the male and female, will often relieve certain nervous diseases, such as migraine, hysteria, epilepsy, simple nervousness and hallucinatory insanity.

In conclusion, I wish to report two cases of epilepsy, one reflex and the other traumatic:

CASE I. Bessie B.; age, 3 years, 10 months; weight, 31 pounds; height, 37 inches; constitution, rather delicate; complexion, fair; hair, brown; eyes, blue.

*Antecedents.*—Parents both living and healthy, offering no hereditary taint of any kind; no history of syphilis or tuberculosis.

*Early History.*—Born at full term. Dentition passed off smoothly, with the exception of some slight gastric trouble, which lasted but a few days; had no convulsions of any kind. She learnt to walk in her tenth month. When two years old she was troubled with ascarides, diagnosticated by their presence in the feces, condition of the child and reflex phenomena. She did not, however, at any time fall into convulsions.

At three years she passed through a severe attack of whooping-cough. Otherwise the child passed its early infancy without any serious disturbance; seemed to be in perfect health, never complaining, always bright, active and cheerful.

*Status Præsens.*—Rather anemic, but well-developed child, offering no particular stigmates, except the one to be described later on. Sensation, mobility and organs of sense unimpaired; organs of secretion and excretion in good working order; no glandular enlargements, and percussion of head is not sensitive. Of late, she has become uneasy, restless, touchy, or, as the mother aptly terms her condition, "she has become nervous."

On March 8, 1888, as the mother was scrubbing the floor, the child, unnoticed, entered the room, stumbled and fell sideways into the tub of boiling water. The outer side of the left leg, from the hip to the foot, was badly burned and blistered. She was put in bed, suffered great pain for two weeks, then made a rapid recovery. At first she was timid about walking, and limped about for some weeks, complaining of pain at the left knee-joint. An examination of the left leg revealed a cicatrix, nearly quadrilateral in shape, situated at the bend of the knee. With the leg extended it measured five centimeters in length and varied from four to five centimeters in width and was locaated on the outer side of the leg, between the outer edge of the patella and the tendon of the biceps muscle. Free movement of the knee-joint is not interfered with. The cicatrix is non-adherent, tender and painful, of a pinkish color and presents one nodosity, cordlike in appearance.

In May, 1888, the mother noticed that at times, while the child was walking, she would suddenly stop, the left leg would stiffen, and the toes, acting as a fulcrum, the whole member would rotate outward, the child's expression would change, but it remained conscious, complaining of a pain, seeming to radiate from the vicinity of the knee-joint. The attacks lasted from one to two minutes. No other phenomena were noticed, and the mother thought the trouble was caused by a faulty shoe.

These attacks occurred at first once daily, and later on twice, continually growing more severe until the extremities became affected. About November, 1888, the child would awaken at night, generally soon after retiring, cry out, the legs would stiffen, then would kick violently for a few moments. The arms remained motionless; no incontinence of urine, no frothing at the mouth and no loss of consciousness. These attacks lasted from three to five minutes, the mother thinks, and would occur nightly for some time, then cease for one or two weeks. Soon these spasms became general. The arms as well as the legs would be seized with tonic, then clonic contractions, followed by loss of consciousness, incontinence of urine, frothing and bleeding at the mouth, pathognomonic symptoms of classical epilepsy. This condition of things existed at the time that I first saw the child, in September, 1889.

I made a diagnosis of epilepsy of reflex character and laid out the following treatment: the cicatrix being too large for excision, I advised painting it daily with collodion. Internally, I prescribed equal parts of the bromides of soda, ammonia and potassium, 5 grains three times daily, and the administration of cod-liver oil. On this treatment the child reacted nobly and the attacks became less severe and less frequent. After two months' treatment the cicatrix appeared more healthy and not painful to pressure. At the end of six months the attacks had disappeared, and since then there has been no recurrence. The bromides were gradually dispensed with, and at the present time (April 5, 1895,) the parents consider the child cured.

CASE II.—*Traumatic Epilepsy.*—W. T., of Attica, N. Y.; age, 24; height, 5 feet 8 inches; weight, 165 pounds; constitution strong and healthy. Nothing in his antecedents or early life is worthy of special consideration. When twelve years of age an accident befell him, on a Fourth of July, which proved to be the cause of his later misfortune. Some boys, having procured the barrel of an old gun, were engaged in discharging it to help celebrate our national holiday. The gun flew into the air and, on descending, struck the patient on the left frontal region of the head. He was carried unconscious to a physician's office, who removed several small bony particles of the skull and sewed up the wound. Suppuration set in, which continued for a few weeks, and the boy seemed to have recovered fully from the effects of the injury. About six years ago, six years after the accident, he began to notice that he felt unusually drowsy, dull and stupid on awakening in the morning. This condition lasted for some time, the cause undiscovered, until he was found in an epileptic attack by his room-mate.

He consulted several physicians without obtaining any relief. In the Fall of 1890, he consulted me, and, without any success, I prescribed bromides. I made a thorough examination, and found a large deep depression over the left frontal bone, the posterior edge extending through the coronary suture. Up to this time he had been able to work hard on a farm, but would have the nocturnal attacks.

On May 2, 1891, I was called to see him, and found him in one of the attacks. He was sitting in a chair, his eyes wide open, glassy, staring, his head drawn to the left, speechless, but not exhibiting any sign of convulsions.

These attacks were growing more frequent, had become diurnal as well as nocturnal, and some radical procedure was demanded. Two days later, I was again called to Attica, and I requested Dr. Mynter to accompany me and operate. On our arrival, we found the patient in the status epilepticus, the attacks being no longer intermittent but continuous, without longer intervals than a couple of minutes. He had been in this condition for thirty hours, one attack of convulsions following the other without the patient regaining consciousness. The pulse was weak and rapid, face cyanotic. A large depression, covered with a tight, whitish, adherent scar, was seen, as described. A large curved incision, convex backwards, was made, the scalp loosened with difficulty from the bone, and an irregular defect of bone found under the scar. The surrounding edges of the bone were much hypertrophied, strongly adherent to the dura and pressing it inward. The dura was loosened all around from the hypertrophied bone, which thereafter was removed all around the defect with cutting forceps.

During the following day he had six attacks, but thereafter he improved rapidly, and he soon returned to his work a well man. No attacks occurred for seventeen months; he was married and felt well, but continued the treatment with bromides. On October 7, 1892, he consulted me again, and stated that the attacks were returning and were becoming daily more frequent. I induced him to enter the Sisters of Charity hospital, where he could be kept under close observation. The attacks, similar to the previous ones, grew daily more severe, and after three days he was again in status epilepticus. Dr. Mynter operated for the second time, opening the wound, detaching the dura from the edges and removed the hypertrophied bony edges. He recovered quickly from the operation and had one spell during the first twenty-four hours. On November 6, 1892, he was discharged, apparently cured, but with the injunction to continue the bromides for an indefinite time. Up to today he has remained free from attacks and has resumed his former occupation.

382 VIRGINIA STREET.

